# Spatial based Expectation Maximizing (EM)

**DOI:** 10.1186/1746-1596-6-103

**Published:** 2011-10-26

**Authors:** M A Balafar

**Affiliations:** 1 Dept of IT, Faculty of Electric and Computer, University of Tabriz, Tabriz, East Azerbaijan, Iran

**Keywords:** Em, Segmentation, Neighbourhood

## Abstract

**Background:**

Expectation maximizing (EM) is one of the common approaches for image segmentation.

**Methods:**

an improvement of the EM algorithm is proposed and its effectiveness for MRI brain image segmentation is investigated. In order to improve EM performance, the proposed algorithms incorporates neighbourhood information into the clustering process. At first, average image is obtained as neighbourhood information and then it is incorporated in clustering process. Also, as an option, user-interaction is used to improve segmentation results. Simulated and real MR volumes are used to compare the efficiency of the proposed improvement with the existing neighbourhood based extension for EM and FCM.

**Results:**

the findings show that the proposed algorithm produces higher similarity index.

**Conclusions:**

experiments demonstrate the effectiveness of the proposed algorithm in compare to other existing algorithms on various noise levels.

## 1. Background

The application of image processing techniques for medical imaging process rapidly increases. Most medical images are stored and represented in softcopy [1]. Ultrasound, X-ray computed tomography, digital mammography and magnetic resonance imaging (MRI) are the most common medical imaging types [2]. MRI can give different grey level for different tissues and various types of neuropathology if its acquisition parameters are adjusted [3].

Data acquisition, processing and visualization techniques facilitate diagnosis. Medical image segmentation plays a very important role in many computer-aided diagnostic tools. These tools could save clinicians' time by simplifying complex time-consuming processes [4]. The main part of these tools is to design an efficient segmentation algorithm. Medical images mostly contain unknown noise [5], in-homogeneity [6] and complicated structures. Therefore, segmentation of medical images is a challenging and complex task. Medical image segmentation has been an active research area for a long time. There are many segmentation algorithms but there is no generic algorithm for a totally successful segmentation of medical images [7].

Clustering methods are common for MRI brain segmentation. Expectation-maximization (EM) and fuzzy c-mean (FCM) are the most popular clustering algorithms. The Gaussian mixture model (GMM) is a popular segmentation method. EM is used to estimate the parameters of this model. FCM and EM only consider the intensity of images and in noisy images, intensity is not trustful [8-10]. Usually, spatially adjacent pixels belong to the same cluster. Many algorithms introduced to make FCM [11-17] and EM robust against noise but nevertheless most of them were and are flawless to some extent. Usually, spatially adjacent pixels belong to the same cluster. Many researchers attempted to incorporate spatial information into FCM and EM to overcome the noise problem. Zhang et. al. [18] proposed a novel Gaussian hidden Markov Random Field (HMRF) model to integrate spatial information into Gaussian model. They used a Markov Random Field-Maximum A Posteriori (MRF-MAP) approach to estimate the model solution. Recently, Tang et al. [19] proposed a neighbourhood-weighted Gaussian mixture model to overcome misclassification on the boundaries and on inhomogeneous regions of MRI brain images with noise. A. R. F. d. Silva [20] proposed two Bayesian algorithms (DPM, rjMCMC) which use Markov chain sampling techniques to find normal mixture models with an unknown number of components. They used algorithms for MRI segmentation and compared performance of their algorithms with published results for two exist Bayesian based MRI brain segmentation methods (KVL [21], MPM-MAP [22]).

González Ballester et al. [23] and Tohka et al. [24] reported a statistical models namely a novel trimmed minimum covariance determinant (TMCD) for the estimation of the parameters of partial volume models to address partial volume averaging.

In order to make Gaussian mixture model more robust against complex tissue spatial layout, Greenspan et al. [25] proposed the parameter-tied, constrained Gaussian mixture model (CGMM) to capture this problem. The mixture model composed of a large number of Gaussians for each tissue is used to capture the complex tissue spatial layout. The Gaussian parameters of a tissue are tied using intensity as global feature. The parameters are learned using the expectation-maximization (EM) algorithm.

In [26], a nonparametric Bayesian model, known as Dirichlet process mixture model (DPMM) is proposed to overcome the limitations of current parametric finite mixture models. The DPMM permits unknown number of components in the mixture and allow robust segmentation of brain with unknown or incomplete specifications.

In [27], local cooperative unified segmentation (LOCUS) approach based on distributed local MRF models for brain segmentation is presented. The volume is partitioned into sub volumes and a set of *local *and *cooperative *Markov random field (MRF) models are distributed. In order to ensure consistency, neighbour local MRFs are estimated cooperatively. The intensity in-homogeneity correction is not required due to precisely fit of Local estimation with the local intensity distribution.

In this paper, a new modification to GMM and EM is introduced by incorporating neighbourhood information into likelihood function and EM steps. The average of neighbour pixels around each pixel is calculated prior to GMM clustering and incorporated in GMM and EM functions beside the pixel value.

The rest of this paper is organized as follows. The standard GMM model and EM segmentation algorithm are presented in Section 2.1. In Section 2.2, proposed modified EM algorithm is described. Also, improvement of segmentation results using use-interaction is presented in section 2.3. Experimental and comparison results are presented in Section 3 and this paper is concluded in Section 4.

## 2. Methods

A modification to GMM is introduced by incorporating neighbourhood information into likelihood function and EM steps.

### 2.1 Standard GMM

The Gaussian mixture model assumes *M *mixed component densities (Gaussian distribution) for each pixel (voxel) with *M *mixing coefficients. Each component is assigned to one target class and the goal is to obtain the class probabilities of each pixel (voxel). The probability distribution of the *j*th component is denoted by *p_j_*(*x_i_*|*θ_j_*), where *x_i _*is pixel *i *in input image and *θ_j _*is the parameter (mean *μ_j _*and covariance matrix ∑*_j_*) of component *j*. The probability distribution each pixel (voxel) can be described as a mixture of probability distributions as follows:

(1) $$\begin{array}{c} p\left(x_{i}|\theta \right)=\overset{M}{\underset{j=1}{\sum }}\alpha_{j}p_{j}\left(x_{i}|\theta_{j}\right)\\ =\frac{1}{\sqrt{\det\left(2\pi \Sigma_{j}\right)}}e^{-{\left(x-\mu_{j}\right)}^{T}\Sigma_{j}^{-1}\left(x-\mu_{j}\right)∕2} \end{array}$$

Where *α_j _*denotes the mixture coefficient with the constraint, $\overset{M}{\underset{j=1}{\sum }}\alpha_{j}=1$ The probability distribution of component *j *is modelled by a Gaussian distribution with mean *μ_j _*and covariance matrix∑*_j_*:

(2) $$p_{j}\left(x_{i}|\theta_{j}\right)=p_{j}\left(x_{i}|\mu_{j},\Sigma_{j}\right)$$

Usually, maximum likelihood (ML) estimation is used to find the parameters. The log-likelihood expression for the parameter *θ *and the image *X *is defined as follows:

(3) $$\begin{array}{c} \text{log}\left(L\left(\theta |X\right)\right)=\text{log}\overset{N}{\underset{i=1}{\prod }}p\left(x_{i}|\theta \right)\\ =\overset{N}{\underset{i=1}{\sum }}\text{log}\left(\overset{M}{\underset{j=1}{\sum }}\alpha_{j}^{t}p_{j}\left(x_{i}|\theta_{j}^{t}\right)\right) \end{array}$$

Finding the ML solution from this equation is difficult. Usually, the expectation-maximization (EM) is used to obtain the parameters. EM steps are demonstrated in the following:

E-step. Bayes' rule is used to obtain the probability of data *x_i _*belong to class *θ_j _*(E-step):

(4) $$p\left(j|x_{i},\theta^{t}\right)=\frac{\alpha_{j}^{t}p_{j}\left(x_{i}|\theta_{j}^{t}\right)}{\overset{M}{\underset{j=1}{\sum }}\alpha_{j}^{t}p_{j}\left(x_{i}|\theta_{j}^{t}\right)}$$

M-step. Probability obtained in E-step is used to obtain mixing coefficient, mean and covariance matrix (M-step):

(5) $$\alpha_{j}^{t+1}=\frac{1}{N}\overset{N}{\underset{i=1}{\sum }}p\left(j|x_{i},\theta^{t}\right)$$

(6) $$\mu_{j}^{t+1}=\frac{\overset{N}{\underset{i=1}{\sum }}x_{i}p\left(j|x_{i},\theta^{t}\right)}{\overset{N}{\underset{i=1}{\sum }}p\left(j|x_{i},\theta^{t}\right)}$$

(7) $$\Sigma_{j}^{t+1}=\frac{\overset{N}{\underset{i=1}{\sum }}p\left(j|x_{i},\theta^{t}\right).\left(x_{i}-\mu_{j}^{t+1}\right){\left(x_{i}-\mu_{j}^{t+1}\right)}^{T}}{\overset{N}{\underset{i=1}{\sum }}p\left(j|x_{i},\theta^{t}\right)}$$

c. EM steps are repeated until convergence.

### 2.2. Modified GMM

The average of neighbour pixels around ${\bar{x}}_{i}$ is calculated prior to GMM clustering. In the likelihood function (Equation 3), distribution value of ${\bar{x}}_{i}$ is added to the distribution value of pixel x_i _as neighbourhood information:

(8) $$\begin{array}{c} \text{log}\left(L\left(\theta |X\right)\right)=\text{log}\overset{N}{\underset{i=1}{\prod }}p\left(x_{i}|\theta \right)=\\ \overset{N}{\underset{i=1}{\sum }}\text{log}\left(\overset{M}{\underset{j=1}{\sum }}\alpha_{j}^{t}\left[\left(1-\beta \right)*p_{j}\left(x_{i}|\theta_{j}^{t}\right)+\beta *p_{j}\left({\bar{x}}_{i}|\theta_{j}^{t}\right)\right]\right) \end{array}$$

The parameter *β *determines the weight of neighbourhood information. Incorporating neighbourhood information improves the performance of segmentation methods in high level of noise, but the blurring effect degrades the performance of them in low noise level. In order to overcome the degrading effect of algorithms in low level of noise, the variance of noise is used to specify the weight of neighbourhood information (*β*). Its value is set to *σ*, where *σ *is the variance of noise. In previous neighbourhood based EM extensions, neighbourhood information is calculated in clustering iteration; but in this algorithm ${\bar{x}}_{i}$ is computed before iteration, thus, the clustering will be faster. An extension of EM named EM-1 is introduced to solve likelihood function. The EM is modified as follows:

a. In Equation 4, distribution value of ${\bar{x}}_{i}$ is added to the distribution value of pixel x_i _as neighbourhood information:

(9) $$\begin{array}{c} A=\left[\left(1-\beta \right)*p_{j}\left(x_{i}|\theta_{j}^{t}\right)+\beta *p_{j}\left({\bar{x}}_{i}|\theta_{j}^{t}\right)\right]\;\\ p\left(j|x_{i},\theta^{t}\right)=\frac{\alpha_{j}^{t}.A}{\underset{j=1}{\sum }\alpha_{j}^{t}.A} \end{array}$$

b. In Equation 6, ${\bar{x}}_{i}$ is added to *x_i _*as neighbourhood information:

(10) $$\mu_{j}^{t+1}=\frac{\overset{N}{\underset{i=1}{\sum }}\left(\left(1-\beta \right)*x_{i}+\beta *{\bar{x}}_{i}\right)p\left(j|x_{i},\theta^{t}\right)}{\overset{N}{\underset{i=1}{\sum }}p\left(j|x_{i},\theta^{t}\right)}$$

c. In Equation 7, the distance of ${\bar{x}}_{i}$ from the component centre is added to the distance of *x_i _*from the component centre as neighbourhood information:

(11) $$\begin{array}{c} d\left(x\right)=\left(x-\mu_{j}^{t+1}\right){\left(x-\mu_{j}^{t+1}\right)}^{T}\\ \Sigma_{j}^{t+1}=\frac{\overset{N}{\underset{i=1}{\sum }}p\left(j|x_{i},\theta^{t}\right).\left(d\left(x_{i}\right)+\beta .d\left({\bar{x}}_{i}\right)\right)}{\overset{N}{\underset{i=1}{\sum }}p\left(j|x_{i},\theta^{t}\right)} \end{array}$$

In MRI, noise behaves as Rician distributed noise. Rician noise approaches Gaussian distribution in high Signal to Noise Ratio (SNR) and Rayleigh distribution in low SNR [28]. Rician distribution in the background is Rayleigh because there is no signal. The Rayleigh PDF of the statistically independent observations is

(12) $$p\left(\left\{O_{i}\right\}\right)=\overset{n}{\underset{i=1}{\prod }}\frac{O_{i}}{\sigma^{2}}e^{-\left(O_{i}^{2}\right)∕\left(2\sigma^{2}\right)}$$

Where *O *is observations and $\sigma_{Noise}^{2}$ is the variance of noise. The variance of noise is obtained by maximizing the log-likelihood of PDF function with respect to variance:

(13) $$\sigma_{Noise}^{2}=\frac{1}{2n}\overset{n}{\underset{i=1}{\sum }}O_{i}^{2}$$

In other words, background pixels are considered as observations (*O) *and the variance of noise is obtained applying equation 13 on background pixels values. For that, the powers of background pixels values are computed and half of the average of resulted values is considered as variance of the noise.

Also, in-homogeneity correction [6] is applied to input image with in-homogeneity pollution and the propose GMM is applied on in-homogeneity corrected image.

### 2.3. Improving Segmentation Results Using User Interaction

Sometimes, due to in-homogeneity, low contrast, noise and inequality of content with semantic, automatic methods fail to segment image correctly. Therefore, for these images, it is necessary to use user interaction to correct method's error [29]. However, robust semi-automatic methods can be developed in which user interaction is minimized.

**Figure 1:**
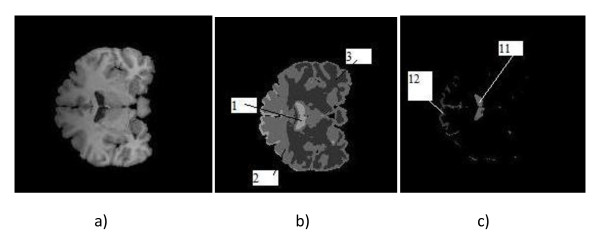
**clustering using user-interaction (a) A real brain volume, (b) its 4 clusters and (c) two sub clusters of Cluster 3**.

Sometimes, segmented image, for example in Figure 1(b), either has pixels from two or more tissues in one cluster (csf and grey matter of brain in cluster number 2) or pixels from one tissue in two or more clusters(white matter in clusters number 2 and 3). For solving this problem, user selects clusters contain several tissues (cluster number 1) to be re-clustered to two sub clusters. Figure 1(c) demonstrates sub clusters of class number 1. The cluster number 1 is clustered to two sub clusters number 11 and 12.

This process continues until user is satisfied. That means quality of segmentation depends on user. Then, to solve problem of several clusters for one tissue, user selects clusters for each tissue (clusters 12 is also selected for grey matter). Steps of this method listed as follow:

1. Input volume is clustered to the n clusters where n is the number of target class (tissues). The output is clustered volume.

2. Under segmentation: If some clusters contain more than one target class (tissue), user selects such clusters to be partitioned more; each user selected cluster is re-clustered to two sub clusters. This process continues till user is satisfied. The output is clustered volume without under segmentation.

3. Over segmentation: If several clusters correspond to one target class (tissue), user selects clusters for each target class. The output is final clustered volume.

## 3. Experimental Results and Discussion

The proposed extension of EM (EM-1) and the existing neighbourhood-based extension of EM [19] (referred as NWEM in this paper for clear understanding) are simulated and tested on the simulated volumes from BrainWeb [30] and real volumes from Internet Brain Segmentation Repository (IBSR) [31].

Moreover, reported results on simulated volumes for existing extensions of EM (DPM, rjMCMC, KVL, MPM-MAP) and existing neighbourhood based extension for FCM (FCM_S [32], FCM_EN [33], FGFCM [34], FLICM [35] and NonlocalFCM [36]) are used to evaluate proposed algorithm.

Also, the reported results on real volumes from IBSR are used to evaluate proposed algorithms. Furthermore, mentioned FCM extensions simulated and tested on real volumes.

The results of algorithms are compared quantitatively to analyse their performance. The neighbourhood size, *N *for proposed algorithm is set to 3 × 3. Three indices (similarity index, false positive ratio and false negative ratio) [37] are used to evaluate the algorithms quantitatively. The similarity index *ρ_i _*of class i is the degree of the class pixels matching between ground truth and segmentation result for the same class. The false positive ratio *r*fp represents extra pixels of class *i *and the false negative ratio *r*fn represents lost pixels of class *i*. They are defined as follows:

(14) $$\rho_{i}=\frac{2|X_{i}\cap Y_{i}|}{|X_{i}|+|Y_{i}|}\;rfp_{i}=\frac{|Y_{i}|-|X_{i}\cap Y_{i}|}{|X_{i}|}\;rfn_{i}=\frac{|X_{i}|-|X_{i}\cap Y_{i}|}{|X_{i}|}$$

where *X_i _*represents class *i *in ground truth and *Y_i _*represents the same class in the segmentation result. Each index for full segmentation results is the average of that index for all classes.

### 3.1. Simulated volumes

The simulated MRI volumes are obtained from BrainWeb. A simulated data volume with T1-weighted sequence, slice thickness of 1 mm and a volume size of 217 × 181 × 181 is used. Non-brain tissues are removed prior to segmentation.

The number of tissue classes in the segmentation is set to three: grey matter (GM), white matter (WM) and cerebrospinal fluid (CSF). All pixels in the image are contributed in segmentation process but in evaluation process, background pixels are ignored following previous works utilized in this paper. In the public databases which have been used in the paper and generally in brain MRI volumes, background pixels have black value. Therefore, cluster with lowest average grey value is considered as background.

**Figure 2:**
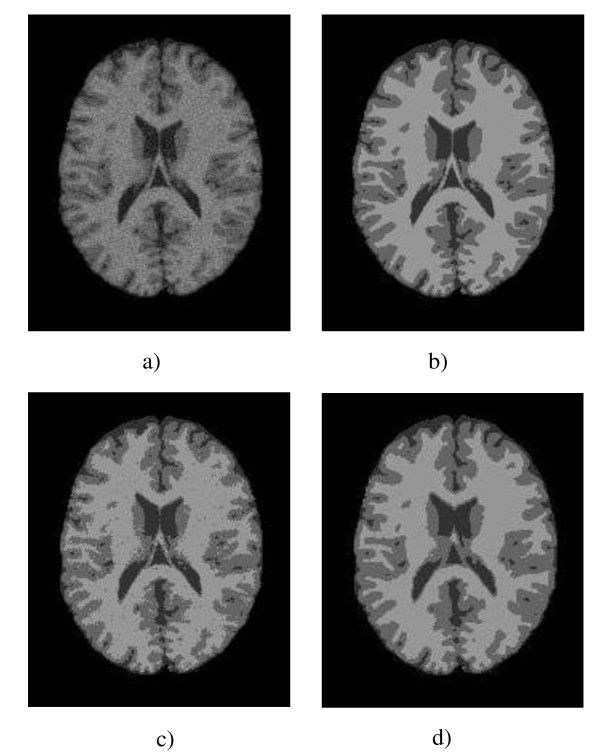
**The segmentation results of applying EM1 and NWEM on a slice of image with 9% Rician noise**. (a) Noisy image, (b) Ground-truth, Segmentation results of (c) NWEM and (d) EM1.

**Figure 3:**
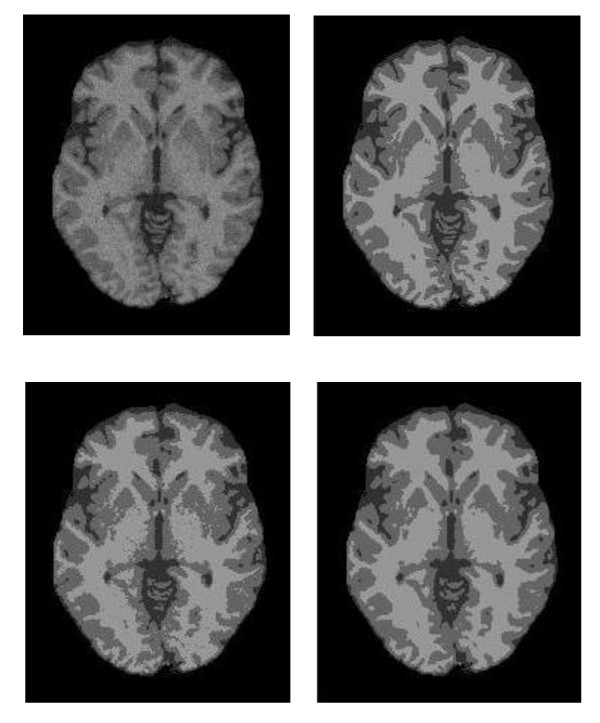
**The segmentation results of applying EM1 and NWEM on a slice of image with 7% Rician noise**. (a) Noisy image, (b) Ground-truth, Segmentation results of (c) NWEM and (d) EM1.

First, EM-1 and NWEM were applied to a slice of T1-weighted brain image corrupted by different noise levels. Figure 2 and Figure 3 show the segmentation results of applying the afore-mentioned algorithms on a T1-weighted normal brain slice in the presence of 9% and 7% rician noise, respectively.

The segmentation results of white matter (WM), grey matter (GM) and cerebrospinal fluid (CSF) are depicted in. (a) is noisy image. (b) is ground-truth. (c) to (d) are the segmentation results of NWEM and EM1, respectively.

From the above qualitative comparison, it was not difficult to find that NWEM was more influenced by the noise in comparison with EM1, in which fewer artefacts were evident, resulting in clearer segmentation result.

**Figure 4:**
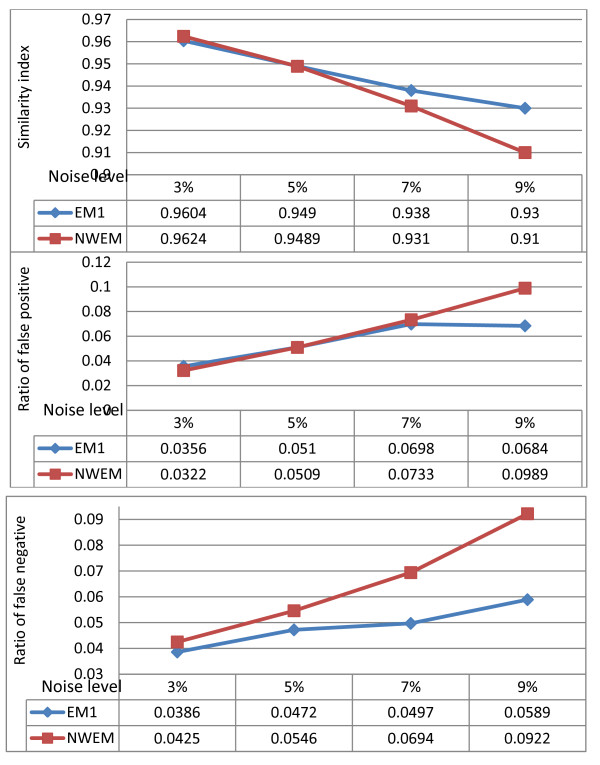
**The average similarity indices *ρ, rfp and rfn *for different noise levels**.

Also, the proposed segmentation algorithm (EM-1) and NWEM are applied to brain volume and average similarity value is used to evaluate them. Figure 4 shows the average similarity indexes *ρ *of mentioned algorithms in different noise levels.

Figure 4 shows that EM-1 produces higher similarity indexes and lower rfp and rfn, meaning that this algorithm produces more accurate segmentation results. The similarity index of EM-1 decreases more slowly than NWEM algorithm when noise level increases. In the same time, the rfp and rfn of EM-1 increases faster than NWEM algorithm.

Both algorithms give similar results, under 5% noise level. However, for more than 5%, EM-1 exhibits much better results than the NWEM algorithm. Incorporating average of neighbourhood information, in clustering process of NWEM, make this algorithm robust against noise but has blurring as side effect. It seems that with increasing noise level more than 5% noise level; this incorporation cannot overcome high level of noise.

**Figure 5:**
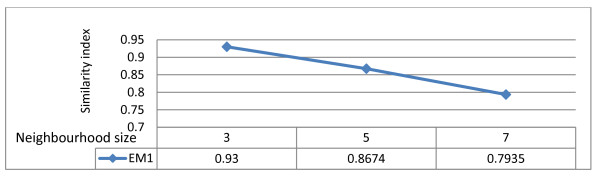
**The average similarity index *ρ *for different neighbourhood sizes on simulated volume with 9% noise**.

Also the effect of different neighbourhood sizes on performance of proposed segmentation algorithm (EM-1) is investigated. Figure 5 shows the average similarity index *ρ *of EM-1 for different neighbourhood sizes on volume with 9% noise. Figure 5 shows that when the neighbourhood size is increased, the similarity index of EM-1 decreases sharply. This means blurring effect in EM-1 depends on neighbourhood size.

**Figure 6:**
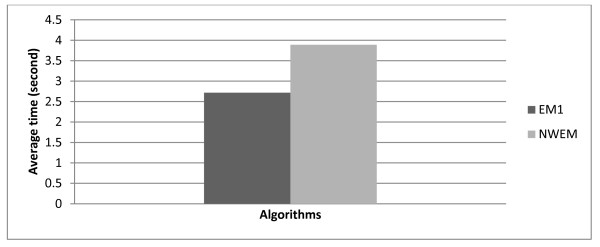
**Average times required to segment a slice using the proposed algorithm (EM1) and NWEM**.

The speed of EM1 and NWEM in segmenting a slice was also investigated. Figure 6 represents the average time required to segment a slice using the mentioned algorithms. Figure 6 shows that EM1 is faster than NWEM. The neighbourhood information in NWEM is calculated in NWEM clustering iteration. Therefore, it is time-consuming.

**Figure 7:**
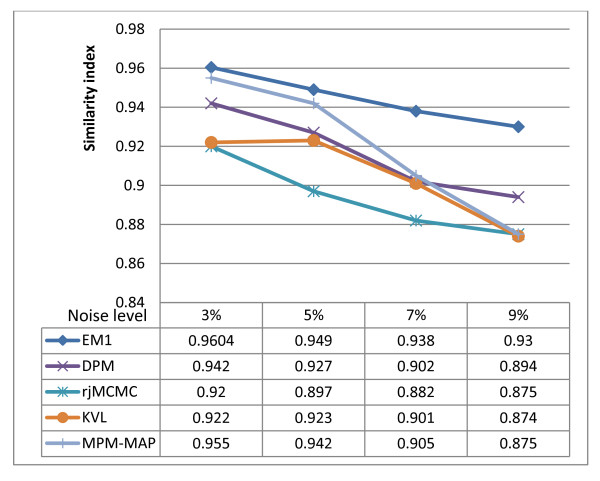
**The average similarity indices *ρ *for different noise levels**.

The proposed segmentation algorithm is also compared with current extensions for EM. The average similarity indexes *ρ *for proposed algorithm (EM-1) and several current extensions for EM (DPM, rjMCMC, KVL and MPM-MAP) are shown in Figure 7. Figure 7 shows that EM-1 produces highest similarity indexes. The proposed segmentation algorithm gives results comparable with the best reported results, in low level of noise. However, for noise levels more than 5%, EM-1 algorithm outperform other competing algorithms and this difference in performance gets more in 9% noise level.

**Figure 8:**
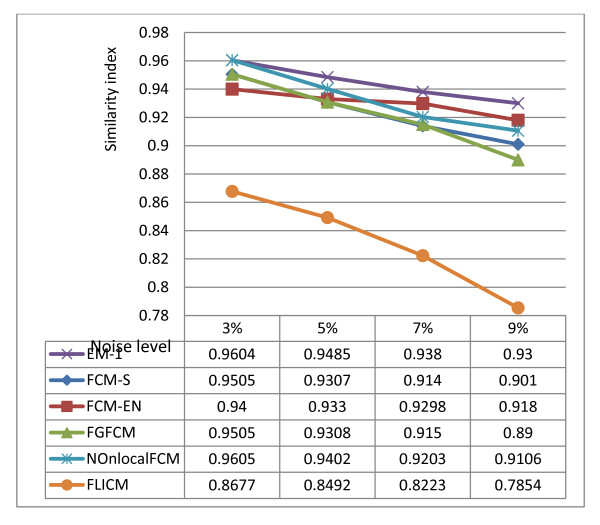
**The average similarity indices *ρ *for EM-1 and FCM extensions in different noise level**.

Also, EM-1 is compared with current existing neighbourhood based extensions for FCM. Figure 8 shows the average similarity indexes *ρ *for EM-1 and FCM extensions (FCM_S, FCM_S1, FCM_EN, FGFCM and FLICM) in different noise levels. At 3% noise level, the results for proposed segmentation algorithm and the best reported result were close. Above 3% noise, EM-1 produces higher similarity index and were the most convincing in segmentation. The superiority of these algorithms increases with increasing in noise level. FLICM shows worst performance it seems it is not suit algorithm for brain volumes.

In [25], the parameter-tied, constrained Gaussian mixture model (CGMM) is applied on image volume from brainweb with different noise levels. Average similarity index for different algorithms with variant noise levels (3%, 5%, 7%, 9%) are: CGMM (0.93, 0.93, 0.92 and 0.895) and KVL (0.925, 0.915, 0.895 and 0.865). The proposed segmentation algorithm outperforms KVL and CGMM.

### 3.2. Real volumes

**Figure 9:**
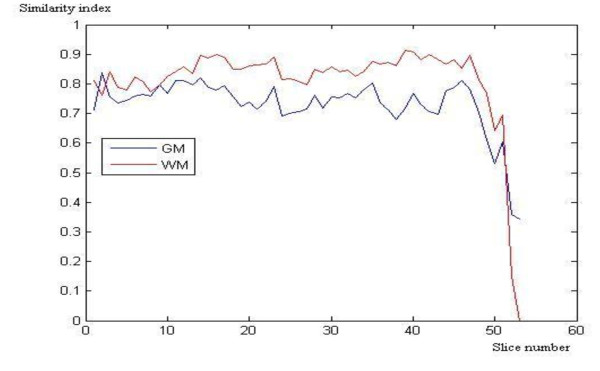
**The similarity index of proposed algorithm when applied for real volume**.

The superiority of our algorithm is also demonstrated on real MRI volumes. The real MRI volumes are obtained from the IBSR by the Centre for Morphometric Analysis at Massachusetts General Hospital. 20 normal data volume with T1-weighted sequence are used. First, proposed algorithm (EM-1) is applied to slices of a real MRI volume with size 256*256*53. The average similarity index *ρ *for volume image is 0.7986. Figure 9 shows the similarity indexes of proposed algorithm (EM-1) for each slice of MRI volume. In almost all slices, the proposed algorithms exhibit better results for WM in compare to results for GM. Better performance of proposed algorithms in WM is due to more simplicity and compactness of WM in compare to GM.

**Figure 10:**
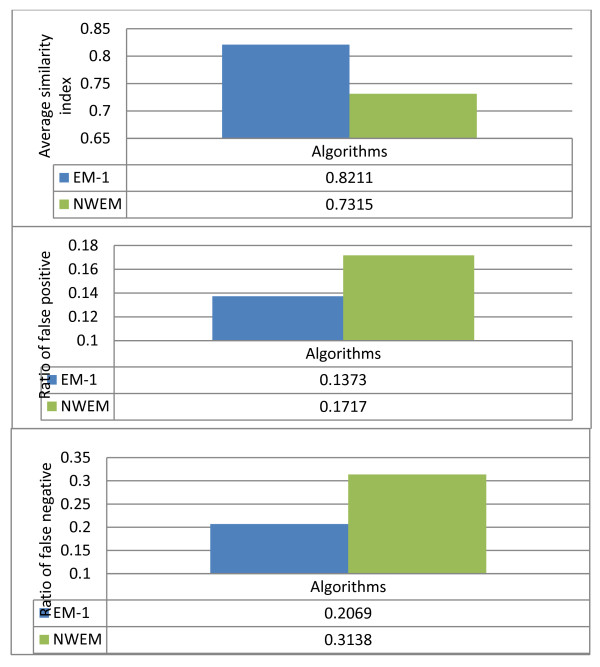
**The average similarity index, rfp and rfn of proposed algorithm when applied on 20 real volumes**.

EM-1 and NWEM are applied to all 20 normal real MRI volumes and average similarity index *ρ *is used to compare the segmentation results, quantitatively. Figure 10 shows the average similarity index, rfp and rfn values of both algorithms for all 20 normal volumes. Figure 10 shows that EM-1 outperforms NWEM. EM-1 produces higher average similarity indexes *ρ *and lower rfp and rfn.

**Figure 11:**
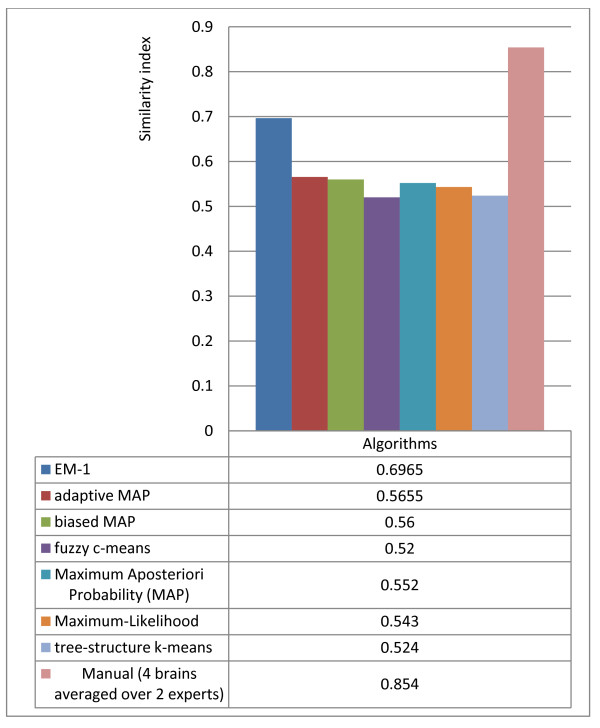
**The average similarity index of different algorithms when applied on 20 real volumes**.

The average similarity index values of proposed algorithm for 20 normal real MRI volumes and EM extensions (reported results in IBSR) are compared. Figure 11 shows the average similarity index values of different algorithms for all 20 normal volumes. Figure 11 shows that the similarity index for proposed segmentation algorithms is higher than competing methods. It can be seen clearly that proposed algorithm has a better performance over reported results, meaning that proposed algorithm produces more accurate segmentation results

**Figure 12:**
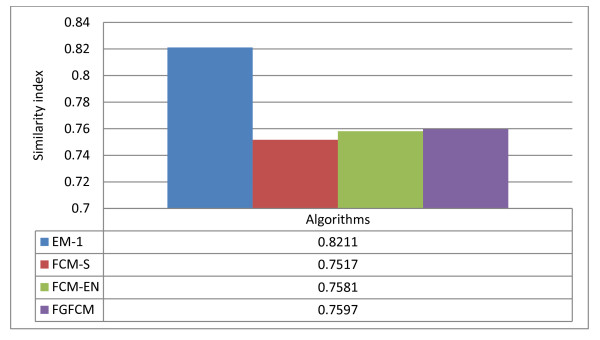
**The similarity index of proposed algorithm and neighbourhood based FCM extensions when applied on 20 real volumes**.

The proposed algorithms are also compared with neighbourhood based extensions for FCM. Figure 12 shows the average similarity indexes *ρ *for proposed algorithm and FCM extensions (FCM_S1, FCM_EN, FGFCM) for all 20 normal volumes.

It can be seen clearly that proposed algorithm has a better performance over FCM extension methods, and produces more accurate segmentation results. FCM extensions also incorporate neighbourhood information in FCM clustering process, but, it seems that incorporating neighbourhood information improves EM more than FCM method.

In [24], a novel trimmed minimum covariance determinant (TMCD) method an extension for Gaussian mixture model is applied on 20 normal image volumes from IBSR. The average jaccard value was 0.6722. The average jaccard values for EM-1 is: 0.695. The similarity index for EM-1 is higher than reported result, meaning that EM-1 produces more accurate segmentation results.

In [25], the parameter-tied, constrained Gaussian mixture model (CGMM) is applied on 18 volumes from 20 normal image volumes (except volume 4-8 and 202-3) in IBSR website. The CGMM results is compared with reported results from the IBSR website, as well as with the Marroquin algorithm [38]. Marroquin's algorithm is an atlas-based Bayesian segmentation algorithm. The CGMM algorithm outperforms other studied methods. Jacc similarity index CGMM was: 0.67. The average jaccard values for EM-1 is: 0.6971. EM-1 outperforms the best reported result which is for CGMM.

In [26], a nonparametric Bayesian model, known as Dirichlet process mixture model (DPMM) is applied on 13 volumes (1_24, 2_4, 5_8, 6_10, 7_8, 11_3, 12_3, 13_3, 15_3, 16_3, 100_23, 110_3 112_2) from the 20 normal T1-weighted brain image volumes from IBSR. The similarity index for DPMM is higher than competing methods. Dice similarity index for DPMM was: 0.7071. The proposed algorithms are applied on the same volumes. The average Dic value for EM-1 is: 0.8219. The similarity index for proposed method is higher than the best reported result which is for DPMM, meaning that proposed method are the most convincing in segmentation.

In [27], local cooperative unified segmentation (LOCUS) approach which is based on distributed local MRF models for brain segmentation is applied on the 20 normal T1-weighted brain image volumes from IBSR. LOCUS-T is compared with published results for SPM5 and FAST. Dic similarity index for different methods are: LOCUS-T = 0.765, SPM5 = 0.81, FAST = 0.765. The average Dic value for EM-1 is: 0.8211. EM-1 outperforms the best reported result which is for SPM5.

**Figure 13:**
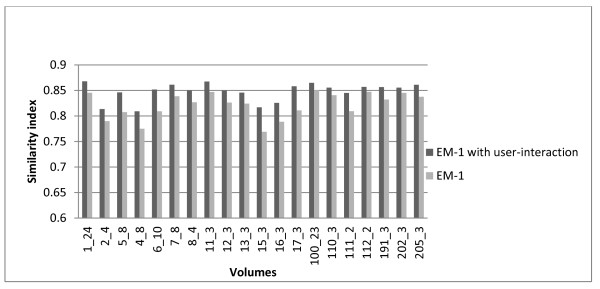
**The similarity index of different algorithms when applied on 20 real volumes**.

Also, improvement of segmentation result using user-interaction is investigated. Proposed algorithms and the same algorithm with user-interaction are applied to all 20 normal real MRI volumes and similarity index *ρ *is used to compare the segmentation results, quantitatively. The average similarity index values of both algorithms for different volume were presented in Figure 13 and Table 1. Figure 13 shows that user-interaction improves performance of proposed algorithm and increases similarity indexes *ρ *in all image volumes.

**Table 1:** The similarity index of different algorithms when applied on 20 real volumes

Volumes Algorithms	EM-1 with user-interaction	EM-1
1_24	0.8678	0.8454

2_4	0.8136	0.7899

5_8	0.8462	0.8076

4_8	0.8091	0.775

6_10	0.8518	0.8091

7_8	0.8613	0.8386

8_4	0.8501	0.8269

11_3	0.8675	0.8471

12_3	0.8498	0.8263

13_3	0.8458	0.8243

15_3	0.8169	0.7691

16_3	0.8256	0.7887

17_3	0.8582	0.811

100_23	0.8649	0.8506

110_3	0.8555	0.8407

111_2	0.8452	0.8095

112_2	0.857	0.8475

191_3	0.8566	0.8322

202_3	0.8555	0.8453

205_3	0.8611	0.8377

## 4. Conclusion

In this paper, an extension of EM has been introduced. In order to overcome the problem of standard EM in the presence of noise, the introduced algorithms are formulated by modifying the equations of the standard EM algorithm which allow the neighbourhood pixels to be incorporated in the labelling of a pixel. Introduced algorithm is tested on simulated MRI volumes, with different noise levels and real volumes. The performance of the existing neighbourhood based EM and FCM algorithms and proposed algorithm are compared qualitatively.

The similarity index, *ρ *is used to evaluate different algorithms. Experiments demonstrate the effectiveness of the proposed algorithm in compare to other existing algorithms on various noise levels in terms of similarity index, *ρ*.

In future, we consider doing research on other kinds of segmentation methods to improve their functionalities. Also, we will analyse the effects of different clustering methods in segmentation of medical images for the diagnosis of abnormal or various important matters in medical images.

## Competing interests

The authors declare that they have no competing interests.

## Authors' contributions

MA performed all works for this paper.
